# A Microfluidic Platform for Screening Gene Expression Dynamics across Yeast Strain Libraries

**DOI:** 10.21769/BioProtoc.4883

**Published:** 2023-11-20

**Authors:** Elizabeth Stasiowski, Richard O’Laughlin, Shayna Holness, Nicholas Csicsery, Jeff Hasty, Nan Hao

**Affiliations:** 1 Department of Bioengineering, University of California San Diego, La Jolla, CA, USA; 2 Department of Chemistry and Biochemistry, University of California San Diego, La Jolla, CA, USA; 3 Department of Molecular Biology, School of Biological Sciences, University of California San Diego, La Jolla, CA, USA; 4 Synthetic Biology Institute, University of California, San Diego, La Jolla, CA, USA

**Keywords:** Yeast library, Protein aggregation, *Saccharomyces cerevisiae*, Yeast, Microfluidics, Microfabrication, High-throughput screening, Gene expression dynamics

## Abstract

The relative ease of genetic manipulation in *S. cerevisiae* is one of its greatest strengths as a model eukaryotic organism. Researchers have leveraged this quality of the budding yeast to study the effects of a variety of genetic perturbations, such as deletion or overexpression, in a high-throughput manner. This has been accomplished by producing a number of strain libraries that can contain hundreds or even thousands of distinct yeast strains with unique genetic alterations. While these strategies have led to enormous increases in our understanding of the functions and roles that genes play within cells, the techniques used to screen genetically modified libraries of yeast strains typically rely on plate or sequencing-based assays that make it difficult to analyze gene expression changes over time. Microfluidic devices, combined with fluorescence microscopy, can allow gene expression dynamics of different strains to be captured in a continuous culture environment; however, these approaches often have significantly lower throughput compared to traditional techniques. To address these limitations, we have developed a microfluidic platform that uses an array pinning robot to allow for up to 48 different yeast strains to be transferred onto a single device. Here, we detail a validated methodology for constructing and setting up this microfluidic device, starting with the photolithography steps for constructing the wafer, then the soft lithography steps for making polydimethylsiloxane (PDMS) microfluidic devices, and finally the robotic arraying of strains onto the device for experiments. We have applied this device for dynamic screens of a protein aggregation library; however, this methodology has the potential to enable complex and dynamic screens of yeast libraries for a wide range of applications.

Key features

• Major steps of this protocol require access to specialized equipment (i.e., microfabrication tools typically found in a cleanroom facility and an array pinning robot).

• Construction of microfluidic devices with multiple different feature heights using photolithography and soft lithography with PDMS.

• Robotic spotting of up to 48 different yeast strains onto microfluidic devices.

## Background

Numerous strain libraries of *Saccharomyces cerevisiae* have been created, including those with genome-wide GFP tagging of open reading frames (Huh et al., 2003), single-gene deletions (Shoemaker et al., 1996), multi-gene deletions (Tong et al., 2001; Kuzmin et al., 2018), and inducible gene expression (Arita et al., 2021). High-throughput screens using these libraries have led to significant advancements in our understanding of genetic function and regulation in budding yeast (Costanzo et al., 2010; Giaever and Nislow, 2014). Recently, Newby et al. constructed a library of strains called yTRAP (yeast transcriptional reporting of aggregating proteins) in which strains have been engineered to express genetic sensors that report on the protein aggregation status of a gene (Newby et al., 2017). This library includes a set of 158 yTRAP sensors for RNA binding proteins as well as several other sensors for different prion proteins such as Sup35, Rnq1, and New1 (Newby et al., 2017). Since protein aggregation as a result of dysregulated proteostasis has been linked to cellular aging in a number of organisms (Lopez-Otin et al., 2013; 2023), we sought to use this library to identify proteins that may be prone to aggregation during yeast replicative aging.

Experiments in microfluidic devices have revealed that single cells proceed along one of two paths during aging, where one path (termed Mode 1 aging) exhibits loss of rDNA chromatin silencing and the other exhibits diminished mitochondrial function and reduced heme levels (termed Mode 2 aging) (Li et al., 2017; Jin et al., 2019; Li et al., 2020; O’Laughlin et al., 2020). Furthermore, we observed a decline in proteostasis in Mode 1 aging cells that coincides with losses of Sir2 activity and rDNA silencing (Paxman et al., 2022). Many RNA-binding proteins contain low complexity domains and are hence aggregation prone. We reasoned that aging or loss of Sir2 activity may lead to aggregation of RNA-binding proteins and hence interfere with their functions. To test this, we developed a high-throughput microfluidic device to screen for RNA-binding proteins that aggregate in response to a sustained loss of Sir2 activity caused by a nicotinamide (NAM) treatment, which induces rDNA silencing loss and mimics the later phases of Mode 1 aging. We used the device to simultaneously monitor the response of multiple yTRAP library strains to NAM treatment and to assess their aggregation state via fluorescence microscopy. Building off of previous high-throughput microfluidic technology developed by our group for screening bacterial libraries of more than 2,000 strains, termed Dynomics (Graham et al., 2020), we adapted this platform for use in yeast by utilizing design principles our group had previously used to trap single yeast cells (Li et al., 2017; Baumgartner et al., 2018). This protocol describes the steps for building and applying a yeast Dynomics microfluidic device that can continuously culture 48 different yeast strains. We used this device to perform a screen of the yTRAP library to identify RNA-binding proteins that aggregate in response to a loss of Sir2 activity (Paxman et al., 2022), however, this microfluidic platform can be used to perform a variety of dynamic screens on different kinds of yeast libraries.

## Materials and reagents

**Materials**

1. SU-8 2002 (Kayaku Advanced Materials, catalog number: Y111029)
2. SU-8 2005 (Kayaku Advanced Materials, catalog number: Y111045)
3. SU-8 2007 (Kayaku Advanced Materials, catalog number: Y111053)
4. SU-8 2075 (Kayaku Advanced Materials, catalog number: Y111074)
5. SU-8 Developer (Kayaku Advanced Materials, catalog number: Y020100)
6. Edge Bead Remover (EBR) PG (Kayaku Advanced Materials, catalog number: G050200)
7. Dow Corning Sylgard 184 kit (PDMS) (Dow, catalog number: 2646340 or Fisher Scientific, catalog number: NC9285739)
8. 0.5 mm biopsy puncher (e.g., World Precision Instrument Reusable Biopsy Punch, catalog number: NC0815069)
9. Scotch tape (3M, catalog number: 50-190-9521)
10. 100 mm (4 inch) Silicon wafers (University Wafer, catalog number: 452)
11. 5 inch × 5 inch × inch glass plate (e.g., McMaster-Carr, catalog number: 8476K15)
12. 1 inch by 3 inch glass slide (e.g., Ted Pella, catalog number: 26007)
13. Plus Plates (Singer Instruments, catalog number: PLU-003)
14. Singer RePads, 96 Short (Singer Instruments, catalog number: REP-002)
15. Singer RePads, 1536 Short (Singer Instruments, catalog number: REP-005)
16. Singer RePads, 6144 Short (Singer Instruments, catalog number: REP-006)
17. SBS-format Acrylic Tool (custom made)
18. Wafer tweezers (e.g., EMS Rubis Style 39S-4, Fisher Scientific, catalog number: 50-239-33)
19. Weigh boats (e.g., Fisherbrand, Fisher Scientific, catalog number: S67090A)
20. 96 well plates, non-tissue culture treated (e.g., Corning, Fisher Scientific, catalog number: 08-772-53)

**Reagents**

1. Tween 20 (e.g., Calbiochem, Millipore Sigma, catalog number: 655204-100 mL)
2. Yeast extract (BD Bacto, catalog number: 21270)
3. Peptone (BD Bacto, catalog number: 211820)
4. Glucose (e.g., Fisher Scientific, catalog number: D16-500)
5. Agar (e.g., Teknova, catalog number: A7774)
6. SC Powder (Sunrise Science Products, catalog number: 1300-030)
7. Yeast nitrogen base (YNB) (BD Difco, Fisher Scientific, catalog number: DF0919-07-3)
8. 10 N NaOH (Fisher Scientific, catalog number: SS255-1)
9. YNB without riboflavin nor folic acid (Sunrise Science Products, catalog number: 1535-250)
10. Helmanex III (Fisher brand, Fisher Scientific, catalog number: 14-385-864)
11. Sulforhodamine B (Invitrogen, catalog number: S1307)

**Solutions**

1. Standard YPD media (see Recipes)
2. Standard SC agar (see Recipes)
3. Microfluidic media (1 L)
4. 2% Helmanex III solution (see Recipes)

**Recipes**

1. **Standard YPD media (1 L)**
  10 g of yeast extract
  20 g of peptone
  20 g of glucose
2. **Standard SC agar (1 L)**
  6.7 g of yeast nitrogen base
  2 g of SC powder
  900 mL of deionized water
  Adjust pH to 5.6 using 10 N NaOH and a pH meter
  Add 16 g of agar and autoclave at 121 °C for 20 min
  Add 20 g of glucose when media has cooled to approximately 50 °C
3. **Microfluidic media (1 L)**
  6.7 g of YNB without riboflavin nor folic acid
  2 g of SC powder
  20 g of glucose
  0.5 mL of Tween 20
4. **2% Helmanex III solution**
  98 mL of deionized water
  2 mL of Helmanex solution

## Equipment

1. Mask Aligner (Optical Associates Inc (OAI) Hybralign Series 200, MDL: 204-092997)
2. Hot plate (e.g., Corning Hot Plate, model: PC-600 D, catalog number: 07-770-109)
3. Profilometer (e.g., Dektak, model: DektakXT)
4. Sonicator (e.g., Branson 8510, model: 8510R-DTH)
5. Oven (e.g., Fisher Scientific, model: 6901)
6. Vacuum desiccator (see Ferry et al. (2011) for vacuum system setup and parts)
7. Singer ROTOR (Singer Instruments, model: ROTOR)
8. Singer Stinger (Singer Instruments)
9. Oxygen Plasma machine (Glow Research AutoGlow plasma system)
10. pH Meter (e.g., Mettler-Toledo AG, SevenCompact pH/Ion S220, catalog number: 01-915-101)
11. Inverted microscope (e.g., Nikon Ti-1 model)

## Software

1. Nikon NIS-Elements (catalog number: MQS31000)
2. Fiji/ImageJ (version 2.1.0/1.53c or another version)
3. RStudio (version 1.3.1093)

## Procedure

- **Photolithography for wafer fabrication**
  See Figure 1 for the device layout and design. The silicon wafer was fabricated using standard photolithography techniques previously described by our group (Ferry et al., 2011). We used standard spin protocols from the SU-8 manufacturer and all layers were spun at the spin speeds listed below for 30 s after an initial 10 s photoresist spreading step. The conduit layer was fabricated using 2002 SU-8 photoresist with a spin speed of 1,000 rpm and had a resulting height of 2.4 μms, the spotting region layer was fabricated using 2005 SU-8 photoresist with a spin speed of 2,600 rpm and had a resulting height of 4.8–5 μms, the HD biopixel layer was fabricated using 2007 SU-8 photoresist with a spin speed of 1,000 rpm and had a resulting height of 12 μms, the minor channel layer was fabricated using 2075 SU-8 photoresist with a spin speed of 4,000 rpm and had a resulting height of 60–65 μms, and the major channel layer was fabricated using 2075 SU-8 photoresist with a spin speed of 2,400 rpm on top of an undeveloped major channel layer including a 1 h edge bead removal step (Lee *et al.*, 2011) at 40 °C on a hot plate using Edge Bead Remover (EBR) PG, resulting in a final height of 145–170 μms. For additional details related to multi-layer photolithography, refer to the accompanying *Bio-protocol* manuscript for Paxman et al., “Fabrication of Microfluidic Devices for Monitoring Yeast Aging” (O’Laughlin et al., 2023).
  **Figure 1.**
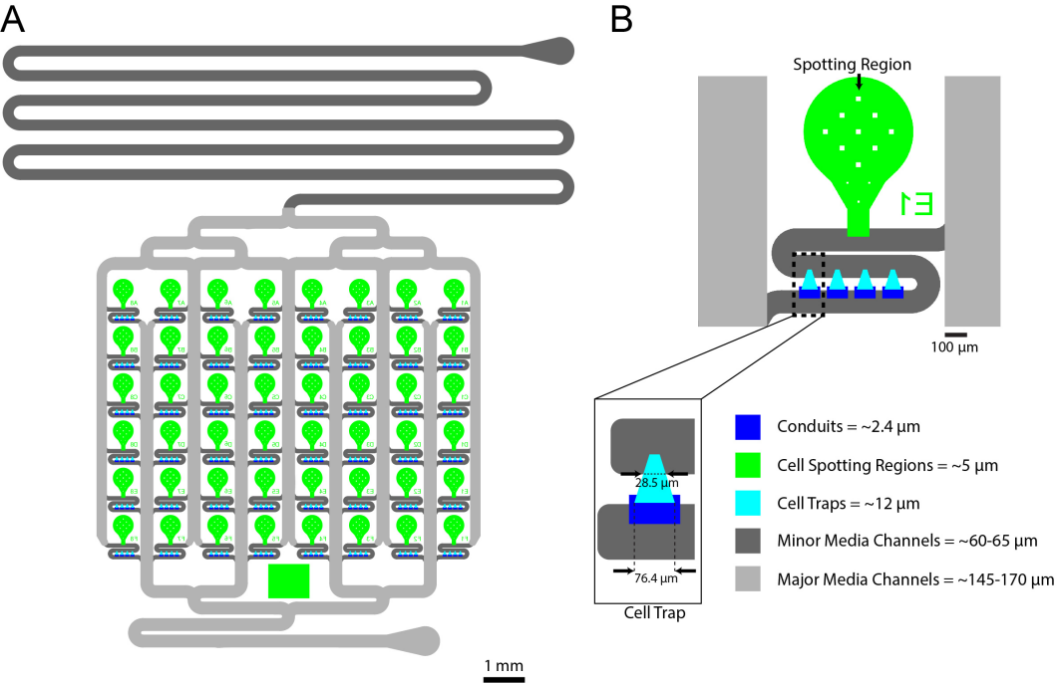
48 Strain yeast microfluidic chip. A. Overall design of the 48-yeast-strain Dynomics microfluidic chip. B. Close-up of single-strain culturing units. The regions robotically spotted by the robot are the green regions (~5 μm tall), the cell trapping areas are the cyan regions (~12 μm tall), the dark-blue areas are the conduits (~2.4 μm tall), the dark-gray features are the minor channels (~60–65 μm tall), and the light-gray features are the major channels (~145–170 μm tall).
- **Soft lithography for fabrication of polydimethylsiloxane (PDMS) devices**
  1. Dispense 70 g of Dow Corning Sylgard 184 elastomer base into a weigh boat and mix with 7 g of Dow Corning Sylgard 184 curing agent. Thoroughly mix together using a clean glass stir rod (approximately 3–5 min).
  2. Place the PDMS mixture in a vacuum desiccator to remove bubbles. The vacuum chamber can be periodically vented to facilitate this process and ensure no overflow of PDMS outside the weigh boat. Remove the mixture from the chamber only when all bubbles have been removed.
  3. Cut out a 16 inch ×16 inch piece of aluminum foil and fold in half twice to produce an 8 inch × 8 inch piece. Place the 5 inch × 5 inch × inch glass plate in the middle of the aluminum foil and gently fold up the edges, ensuring that the foil does not tear, to create a bowl or dish around the plate. Overlap the aluminum foil over the edges of the glass to minimize PDMS leaking underneath the glass dish.
  4. Place the photopatterned silicon wafer in the center of the glass plate with surrounding aluminum foil walls and pour on the bubble-free PDMS mixture.
  5. Degas the PDMS again by placing the wafer in a vacuum desiccator, ensuring that it is level. Remove only once all bubbles have been removed (approximately 30 min–1 h) (Figure 2A).
    **Critical step:** It is vital that the vacuum desiccator is completely level so that a flat PDMS device is produced. This is a requirement for uniform cell spotting in subsequent steps.
  6. After all bubbles are cleared, remove the wafer from the vacuum chamber. If needed, use two pipette tips to re-center the wafer. Carefully push down on opposite sides of the wafer to ensure it is in even contact with the glass plate and to push out any PDMS that may have seeped underneath the wafer (Figure 2B).
  7. Place the wafer stack into a level oven and bake at 95 °C for 1 h.
    **Critical step:** The oven must be level in order to obtain a flat PDMS device. Note that PDMS shrinks slightly during baking, and 95 °C must be used as the cell traps are designed to accommodate the amount of shrinkage at this temperature.
    **Figure 2.**
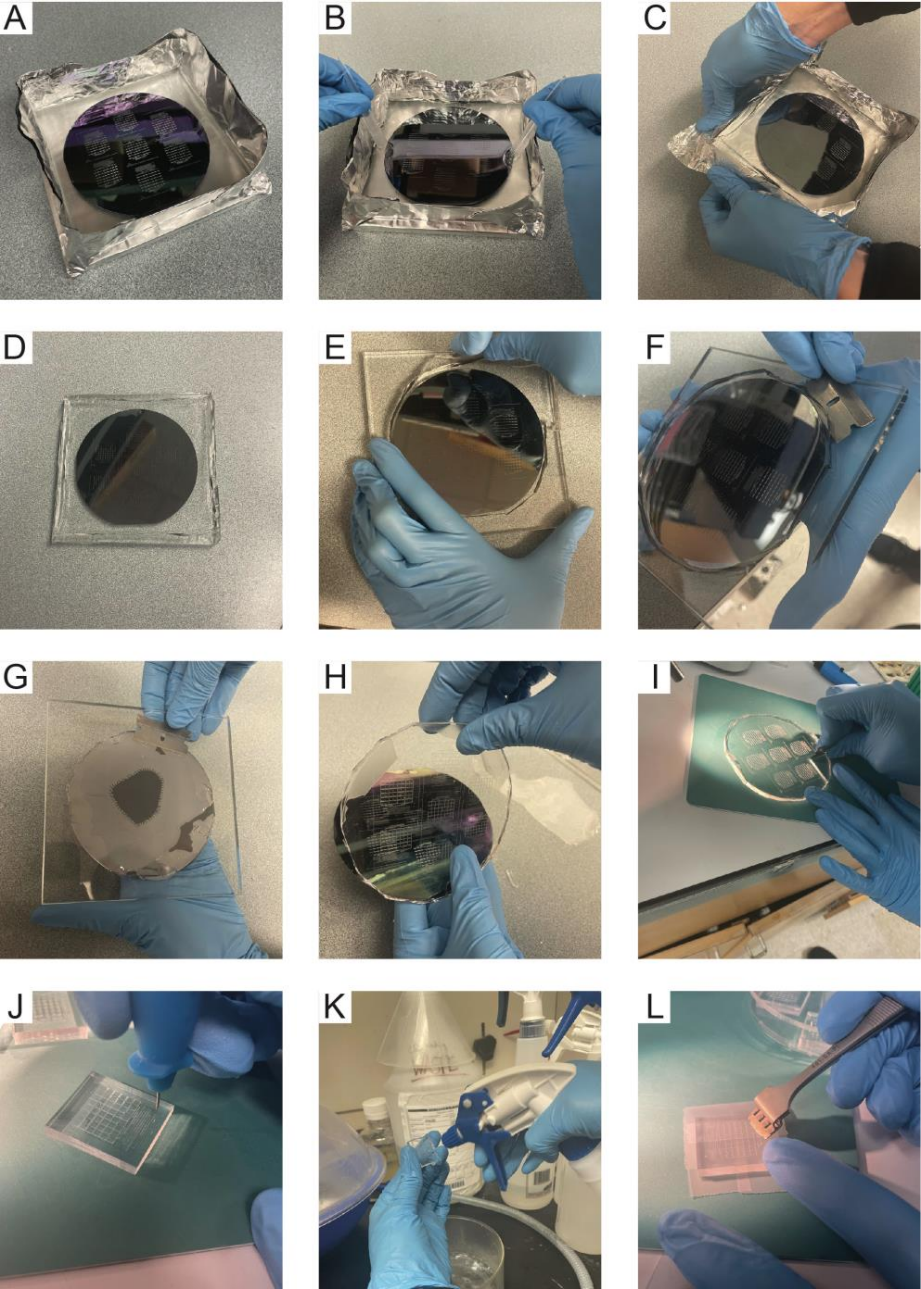
Soft lithography with polydimethylsiloxane (PDMS) for making microfluidic chips. A. PDMS is poured onto the silicon wafer in an aluminum foil boat. B. The wafer is centered using pipette tips. C. After baking, aluminum foil is removed from the wafer edges first. D. Aluminum foil fully removed from wafer. E. Removing PDMS around the wafer. F. Using a razor blade to detach PDMS from the front surface of the wafer. G. Removing PDMS from the back of the wafer. H. Peeling PDMS off of the wafer. I. Cutting out individual chips from the PDMS. J. Punching the cell loading and waste ports from the PDMS. K. Cleaning PDMS chips with 70% ethanol. L. Cleaning chips with Scotch tape.
  8. Peel off the aluminum foil from the wafer stack and remove any excess PDMS around the wafer using a razor blade (Figure 2C–2E).
  9. Gently slide a razor blade horizontally between the wafer and glass plate and then remove it. Repeat this around the circumference of the wafer until the wafer separates from the glass plate (Figure 2F).
    **Critical step:** The razor blade must slide horizontally between the glass and the wafer. Wafers are extremely fragile; if the razor blade is angled, then the wafer will break.
  10. Using a razor blade, remove any excess PDMS from the bottom of the wafer (Figure 2G). Peel the PDMS off of the feature side of the wafer in the direction of the major channels (Figure 2H).
  11. Place the PDMS on a cutting mat with the feature-side up to keep the PDMS clean. Using a razor blade, cut out each PDMS device (Figure 2I). Punch out the inlet and outlet channels with an 0.5 mm biopsy puncher (Figure 2J).
  12. Rinse devices with 70% ethanol and blow dry with compressed air or pressurized nitrogen gas (Figure 2K).
  13. Use scotch tape to clean devices. Clean the feature side four times and the non-feature side twice. Use forceps to gently press the tape into the features to remove all debris (Figure 2L). Leave a fresh piece of tape on each side to keep the devices clean or store them for later use. Note that PDMS devices can be made well in advance of experiments and stored in this condition; however, it is best practice to use PDMS that is no more than two months old.
- **Cleaning glass slides**
  1. Sonicate glass slides in a 2% Helmanex III solution for 30 min at 40 °C.
  2. Rinse glass slides with deionized water, rubbing them with a clean nitrile glove.
  3. Completely dry the glass slides with pressurized nitrogen gas and ensure that no streaks are visible.
    **Pause point:** Clean glass slides can be stored in a clean, dust-free Petri dish until used.
- **Cell preparation and device loading using singer ROTOR agar plate preparation**
  1. Dispense 42 mL of SC agar into Singer Plus Plates on a level surface.
    **Critical step:** The plates must be flat to ensure full cell transfer to plates and microfluidic devices.
  2. Allow plates to dry on the benchtop with the lids covered for 48 h before adding Parafilm and putting in a 4 °C fridge. Plates can be stored at 4 °C for up to six months.
- **Cell preparation (3–4 days)**
  1. Fill each well of a 96-well plate (e.g., 200–300 μL) with a strain from the yTRAP library and allow cells to grow in Standard YPD media overnight in a 30 °C shaker.
  2. Using the Singer ROTOR, spot the liquid plate onto an SC agar Plus Plate. Use the default pinning settings for both the source and target plates. Allow yeast strains to grow up on the agar plates for two days at 30 °C.
*Note: The Singer robot is compatible with plates of 96, 384, 1536, and 6144-density. The 6144 density has a 1.125 mm spacing between arrays, matching the design of our microfluidic device. While the Singer robot can array directly from 96 to 6144 density, the number of cells deposited is variable, and 6144 plates cannot be stored in the fridge for reuse. Therefore, we array strains from a 96-density to 4×1536 density plates that are combined to the microfluidic-matching 6144-density. The number of strains placed onto the 1536-well plates is dependent on the size of the microfluidic device. In this context, we array 12 strains per each 1536 plate including replicates. Furthermore, we array up to eight devices per plate. See Figure 3 for a summary of the arraying procedure.*
  3. Using the Singer Stinger single colony picker, re-array the 96-agar plate onto a set of four 1536-density SC agar plates that matches the array of the microfluidic device(s).
  4. Grow cells overnight at room temperature.
    **Pause point:** 1536-density plates can be stored in the fridge and continually used as source plates for up to six months.
    **Figure 3.**
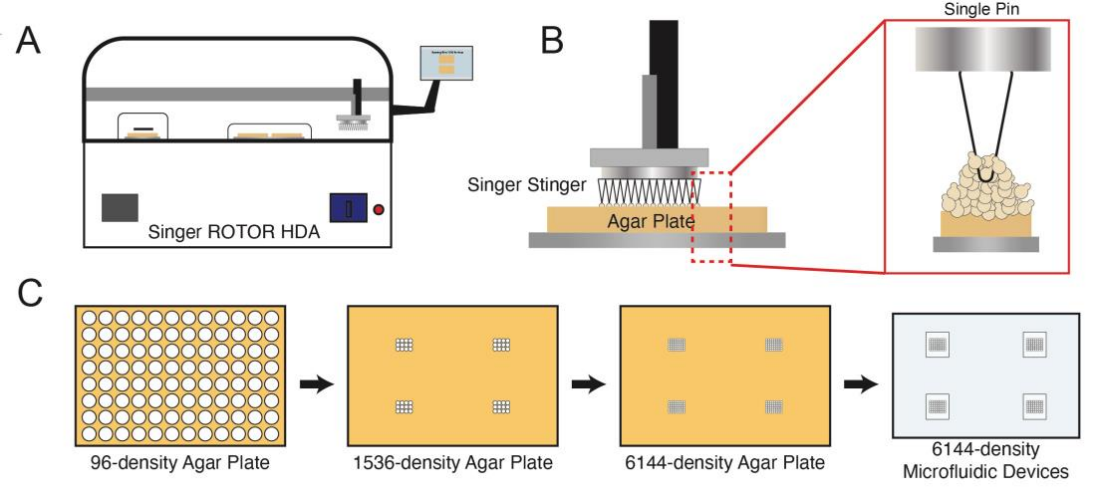
Overview of robotic spotting process. A. Plates are spotted using the Singer ROTOR array pinning robot. B. Individual pins allow strains to be arrayed in rectangular grids on agar plates. C. Arraying process for the Dynomics microfluidic device.
- **6144-density plate and acrylic tool preparation**
  1. Using the Singer ROTOR, replicate the four 1536-density agar source plates onto four 1536-density SC agar source plates using the *Replicate* program. Grow the plates at 37 °C overnight.
    **Critical Step:** *S. cerevisiae* must be heat-shocked overnight at 37 °C in order to be viable when revived on the microfluidic device.
  2. Using the Singer ROTOR, combine the four 1536-density agar source plates onto one 6144-density SC agar plate using the 1:4 array program and the pinning settings listed in Table 1.
    **Table 1.** Arraying parameters for spotting yeast

	Pinning pressure (%)		Pinning Speed (mm/s)		Pinning overshoot (mm)	
	**Source**	**Target**	**Source**	**Target**	**Source**	**Target**
**1536 agar to 6144 agar**	58	64	19	10	2	1
**6144 agar to acrylic**	50	100	10	10	0.6	0.6
**6144 agar to microfluidic device for *S. cerevisiae***	50	64	10	10	0.6	0.6
  3. Using the *Replicate* program on the Singer ROTOR and the pinning setting listed in Table 1, spot cells from 6144-density target plate onto the clean acrylic alignment tool. These cells will be used as alignment markers for the PDMS device.
- **Aligning the PDMS to the acrylic tool**
  1. Using a mask aligner, set the acrylic tool on top of the mask holder with the alignment cells facing up. Bring the cells of one device into focus on the optics.
  2. Remove the scotch tape from one PDMS device, avoiding touching the feature side of the device.
  3. Gently place the PDMS device on top of the alignment cells, feature-side up, such that the center of the spotting regions is centered over the cells.
  4. Place tape on top of the PDMS, pressing the PDMS down to ensure adhesion of the PDMS onto the acrylic tool.
  5. Repeat this process for each device on the acrylic tool.
- **Oxygen plasma exposure**
  1. Expose the clean 1 inch × 3 inch glass slide and the PDMS acrylic stack to 30 W of oxygen plasma for 30 s.
  2. Blow any dust off the glass slide and PDMS acrylic stack with compressed nitrogen.
- **Cell preparation for *S. cerevisiae* spotting**
  1. Using the Singer ROTOR, combine the four heat-shocked 1536-density SC agar source plates onto one 6144-density SC agar plate using the *1:4 Array* program and the pinning settings listed in Table 1.
    **Critical Point:** This plate should be used immediately to spot cells onto the oxygen plasma–exposed PDMS device.
- **Loading and bonding the device**
  1. Using the Singer ROTOR and the parameters listed in Table 1, spot the cells from the 6144-density SC agar plate to the oxygen plasma–exposed PDMS acrylic stack.
  2. Peel the spotted PDMS off the acrylic piece and gently place it face down on the center of the oxygen plasma–exposed glass slide. Gently tap the top of the PDMS, ensuring that the device bonds to the glass.
  3. Incubate the device at 37 °C for at least two hours.
  4. See Figure 4 for information on how spotted cells load into the cell trapping chambers that are used for experimental analysis.
    **Critical Point:** Spotted chips should be wetted with media and set up on the same day to ensure complete cell revival.
    **Figure 4.**
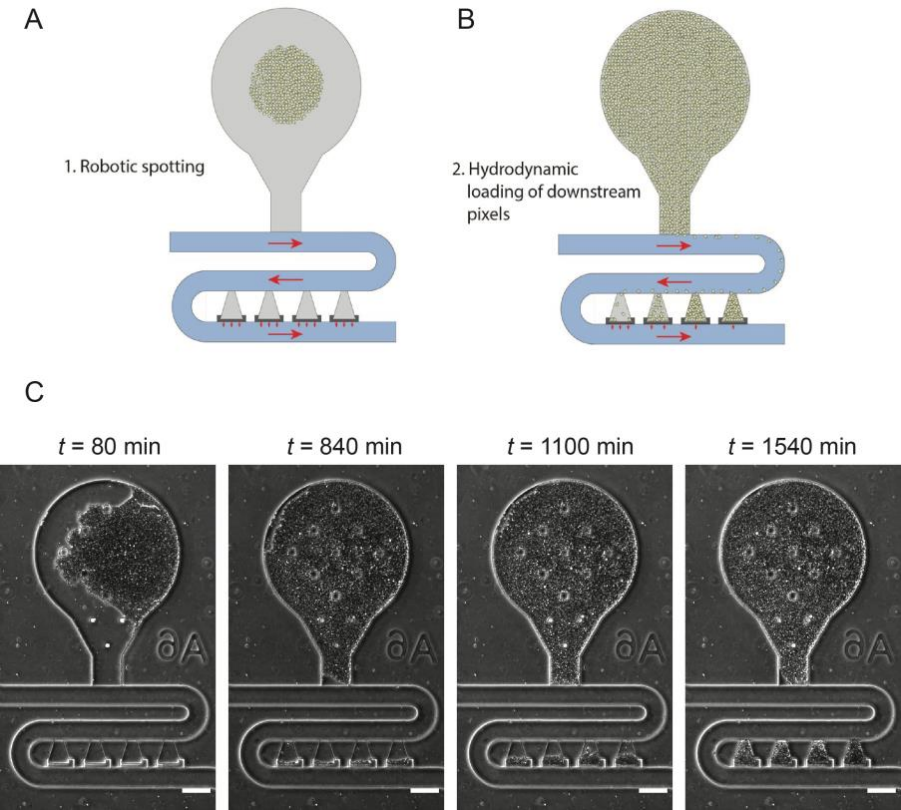
Loading of robotically spotted cells into downstream traps. A. Each of the 48 spotted strains resided in a unique bulb-shaped region of the device (gray region). B. During experiments, as cells grow up in the bulb region, they flow into the four downstream traps, which we refer to as hydrodynamic biopixel traps. Cells in these traps are used for experimental analysis. Red arrows indicate the direction of media flow. C. Filling of downstream biopixel traps during experiments. Scale bar = 100 μm.
- **Setting up the microfluidic experiment**
  1. Place the spotted chip into the vacuum desiccator for 20 min.
  2. Wet the chip by adding at least 20 µL of microfluidic media to both the inlet and outlet port.
*Note: Additional details for setting up microfluidic experiments from this point forward are explained at length in (Ferry et al., 2011). Further, additional details about the experimental and imaging parameters used for the yTRAP library screen are given in Paxman et al. (2022).*

## Data analysis

**Figure 5.**
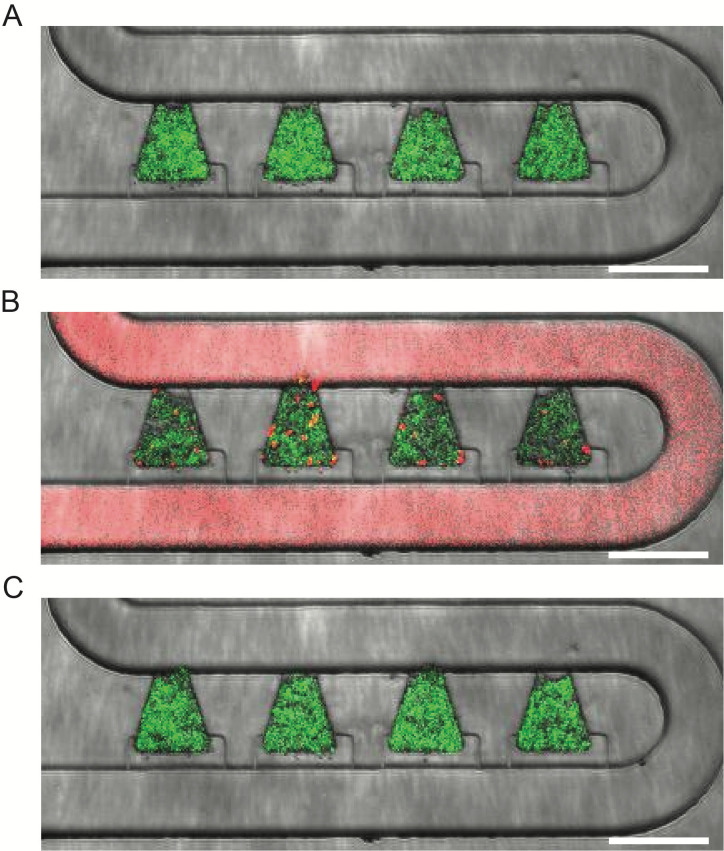
Example response of the SCP160 strain to nicotinamide (NAM). A. Strain fluorescence before NAM exposure. B. Strain fluorescence during NAM exposure. Sulforhodamine B dye was added to the NAM-containing media (1:10,000 ratio) to aid in visualization. C. Strain fluorescence after NAM exposure. Scale bar = 100 μm.

**Figure 6.**
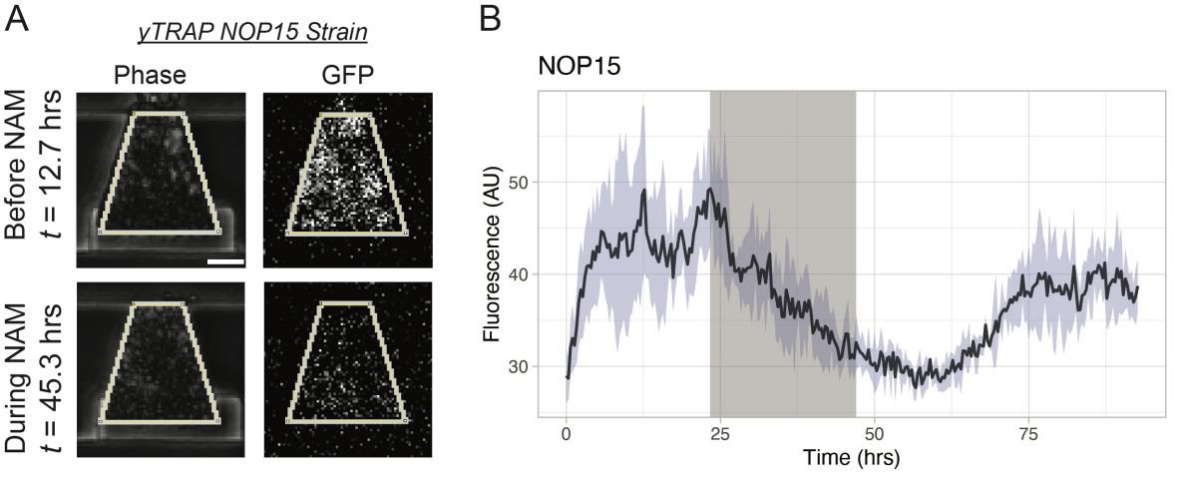
Analysis of yTRAP strain responses to nicotinamide (NAM). A. Extraction of the fluorescence signals in the GFP channel using manually drawn regions of interest in the corresponding frame in the Phase channel. The NOP15 yTRAP strain is used as an example. Scale bar = 25 μm. B. Mean NOP15 yTRAP GFP signal across all four biopixels. Light-blue shaded area represents the standard deviation. Gray region of the plot represents the NAM exposure window.

Example images of yTRAP strains growing in the device and responding to nicotinamide (NAM) exposure are shown in Figure 5. For analysis, background subtraction using a rolling ball radius of 50 pixels was done in ImageJ on the GFP channel, which contained the fluorescence signal of each yTRAP strain. Using the phase channel as a guide, biopixels were manually outlined using the polygon tool in ImageJ and used to create regions of interest (ROIs). These ROIs were then used to extract fluorescence information from the corresponding biopixel in the GFP channel (Figure 6A). Fluorescence signals from all four biopixels were averaged together to create a response curve for each strain (Figure 6B).

To determine yTRAP strains that exhibited aggregation during the NAM induction, the average response curves for each strain were normalized between 0 and 1. Normalized traces were smoothed using the loess() function in R, with the “span” parameter set to 0.2. Using these normalized traces, we then approximated the first derivative for each fluorescence trace by calculating the forward difference. We then filtered the dataset to only include those strains that displayed both a decreasing fluorescence signal for more than half of the NAM induction as well as at least a 75% decrease in fluorescence during the induction (as measured relative to the average fluorescence value of that strain 3 h before the NAM induction). From this list, images were manually verified to ensure decreases in fluorescence were not affected by cell washout from the traps or similar artifacts. This produced a list of 15 strains for further analysis.
